# Cost-effectiveness of a centrifugal-flow pump for patients with advanced heart failure in Argentina

**DOI:** 10.1371/journal.pone.0271519

**Published:** 2022-08-01

**Authors:** Andrea Alcaraz, Andres Pichon-Riviere, Carlos Rojas-Roque, Juan Martín González, Daniela Prina, Germán Solioz, Federico Augustovski, Alfredo Palacios

**Affiliations:** Health Technology Assessment and Health Economics Department, Institute for Clinical Effectiveness and Health Policy (IECS), Buenos Aires, Argentina; Universidad Nacional Autonoma de Nicaragua Leon, NICARAGUA

## Abstract

**Background:**

Centrifugal-flow pumps are novel treatment options for patients with advanced heart failure (HF). This study estimated the incremental cost-effectiveness ratio (ICER) of centrifugal-flow pumps for patients with advanced HF in Argentina.

**Methods:**

Two Markov models were developed to estimate the cost-effectiveness of a centrifugal-flow pump as destination therapy (DT) in patients with contraindication for heart transplantation, and as bridge-to-transplant (BTT), with a lifetime horizon using the third-party payer Social Security (SS) and Private Sector (PS) perspectives. Clinical, epidemiological, and quality-adjusted life years (QALY) parameters were retrieved from the literature. Direct medical costs were estimated through a micro-costing approach (exchange rate USD 1 = ARS 59.95).

**Results:**

The centrifugal-flow pump as a DT increased the per patient QALYs by 3.5 and costs by ARS 8.1 million in both the SS and PS, with an ICER of ARS 2.3 million per QALY. Corresponding values for a centrifugal-flow pump as BTT were 0.74 QALYs and more than ARS 8 million, yielding ICERs of ARS 11 million per QALY (highly dependent on waiting times). For the 1, 3, and 5 GDP per QALY thresholds, the probability of a centrifugal-flow pump to be cost-effective for DT/BTT was around 2%/0%, 40%/0%, and 80%/1%, respectively.

**Conclusion:**

The centrifugal-flow pump prolongs life and improves the quality of life at significantly higher costs. As in Argentina there is no current explicit cost-effectiveness threshold, the final decision on reimbursement will depend on the willingness to pay in each subsector. Nevertheless, the centrifugal-flow pump as a DT was more cost-effective than as a BTT.

## Introduction

Heart failure (HF) is a chronic progressive and incurable condition that occurs when the heart cannot pump or fill with blood adequately. HF affects more than 23 million individuals worldwide [1] and its mortality rate continues to be high [2]. Although the prognosis of chronic HF has improved with the implementation of evidence-based therapies [3–9], existing therapies delay, but not reverse, the progression of the disease. In the advanced stages, HF patients have an extremely high annual mortality rate—nearly 70% of patients die at 1-year. In addition, frequent hospitalizations and the need for multiple pharmacological treatments and interventions [10], markedly deteriorate their health-related quality of life (HRQoL) and increase healthcare costs [11]. As the prevalence of HF increases due to population aging [12] and the increase in predisposing diseases such as diabetes, obesity, and hypertension [13], the resulting economic toll to health systems can become unaffordable. Estimates suggest that the total costs of HF management are expected to increase by 127% between 2012 and 2030 [14]. Therefore, decision making based on effectiveness and cost is required to provide patients with HF with improved HRQoL and tackle the rising health care costs to health systems.

A heart transplant is the gold standard treatment for patients with advanced HF, but this option depends on the supply of donor hearts. Certainly, the shortage of donor hearts is the leading limiting factor that disrupts heart transplantation as an intention to treat principle, and it is also a factor that increases death rate in patients on the waiting list for heart transplantation [15,16]. Within this context, the left ventricular assist device (LVAD) as a bridge to transplant (BTT) is an alternative treatment for patients with advanced HF [17]. LVAD as BTT consists in surgically implanting a mechanical pump to support left ventricular function until a donor heart becomes available. The 1-year survival rate of these patients is similar to that of heart transplantation [18]. In addition, a randomized controlled clinical trial for transplant-ineligible patients exhibited higher survival rates in the LVAD group as a destination therapy (DT) than as medical treatment [19].

Previous economic evaluations had revealed that the incremental cost-effectiveness ratio (ICER) for LVAD as BTT or DT did not meet the threshold for cost-effectiveness in high-income countries [20]. However, little is known to date about the cost-effectiveness of the novel centrifugal-flow pump. Compared to the previous LVAD (known as continuous flow device), the centrifugal-flow pump is associated with a less frequent need for pump replacement and with a superior survival free of disabling stroke or reoperation to replace or remove a malfunctioning device [21]. Thus, this device may hold promise for a cost-effective treatment of HF.

In Argentina the health system is fragmented and segmented into three large sectors: the public, social security, and private sectors. Nearly 36% of the population (16 million) receives healthcare in the public sector. The social security sector is the largest and provides health coverage to 60% (26 million) of the population. Within that sector, the Obras Sociales Nacionales (OSNs), mostly composed of workers within the same labor activity and their core relatives, cover 14 million individuals. The Obras Sociales Provinciales (OSP) include all public employees and provide coverage to 7 million people. The remaining 5 million people (composed of elderly and people with disability) are covered by a nationwide social health insurance fund for retired workers. The private sector covers approximately 6 million individuals, composed of 4 million people that come from the OSNs contracting private supplementary plans, while the remaining 2 million are enrolled on an individual basis through direct and voluntary payment [22,23].

The prevalence of HF in Argentina is between 1% and 1.5%, but it increases up to 8-fold in adults 65 years old or older [24]. Moreover, the mean waiting time on heart transplant lists is relatively low, near three months for candidates in urgent condition [25]. Consequently, it is important to use cost-effectiveness evidence on HF treatment to allocate the scarce resources to improve patient health. This study aimed to estimate cost-effectiveness measured through the incremental cost per QALY of the centrifugal-flow pump, as both BTT and DT, for patients with advanced HF in Argentina.

## Methods

### Decision model

We developed two-stage transition (i.e. Markov) models in Microsoft Excel® (Microsoft Corp. Redmond, WA) to determine the cost-effectiveness of using the centrifugal-flow pump (HeartMate 3). Two well-defined populations were followed using a lifetime time horizon: i) Model 1 compared patients with contraindication for heart transplant and indication for a centrifugal-flow pump as DT versus patients receiving standard medical treatment (hospitalized inotrope-dependent patients or outpatient medical treatment); ii) Model 2 compared patients with an indication for heart transplant plus centrifugal-flow pump as a BTT versus patients receiving heart transplant directly.

For both models, two third-party payers’ perspectives were analyzed: the social security (SS) perspective and the private sector (PS) perspective. In Argentina, the SS is the dominant health sector and provides health coverage to 60% of the population, while the PS provides coverage to 5% of the population [22]. Following the economic evaluation guidelines for countries that belong to MERCOSUR [26], costs and health benefits beyond one year were discounted at a rate of 5%. In both models, the primary outcome was the ICER, expressed as cost per quality-adjusted life-year (QALY) gained. We used the Consolidated Health Economic Evaluation Reporting Standards (CHEERS) Statement to report the findings [27].

Due to the heterogeneity in the willingness to pay among health subsectors of Argentina and the lack of a specific explicit cost-effectiveness threshold, the decision rule to define the cost-effectiveness threshold was assessed within a range going from one (ARS $ 700,473) to five (ARS $ 3,502,363) gross domestic product (GDP) per-capita per QALY gained. The range of the decision rule was defined based on previous economic evaluations published in Argentina [28–32] and the willingness-to-pay threshold of 5 GDP was explored, since the indication for a centrifugal-flow pump is recommended for end-of-life care in the case of a disease associated with short life expectancy that would be extended thanks to the device. The consideration of a higher willingness-to-pay has been contemplated by the National Institute for Health and Care Excellence (NICE) in the United Kingdom upon establishing the criteria for the appraisal of end-of-life treatments [33], and has been used in a recent study in Argentina [30].

### Patient characteristics

Both models were applied to a hypothetical population of 1000 adult patients diagnosed with advanced HF in functional class (FC) III / IV, according to the classification of the New York Heart Association (NYHA) [34], with body surface area > 1.2/m^2^, left ventricular ejection fraction ≤25%, inotrope-dependent or with poor response to optimal outpatient medical treatment or with short-term ventricular assistance (for example, intra-aortic balloon pump). This population corresponds to categories 1 to 4 of the INTERMACS classification, for patients with advanced HF [35].

### Model structure

**Fig 1:**
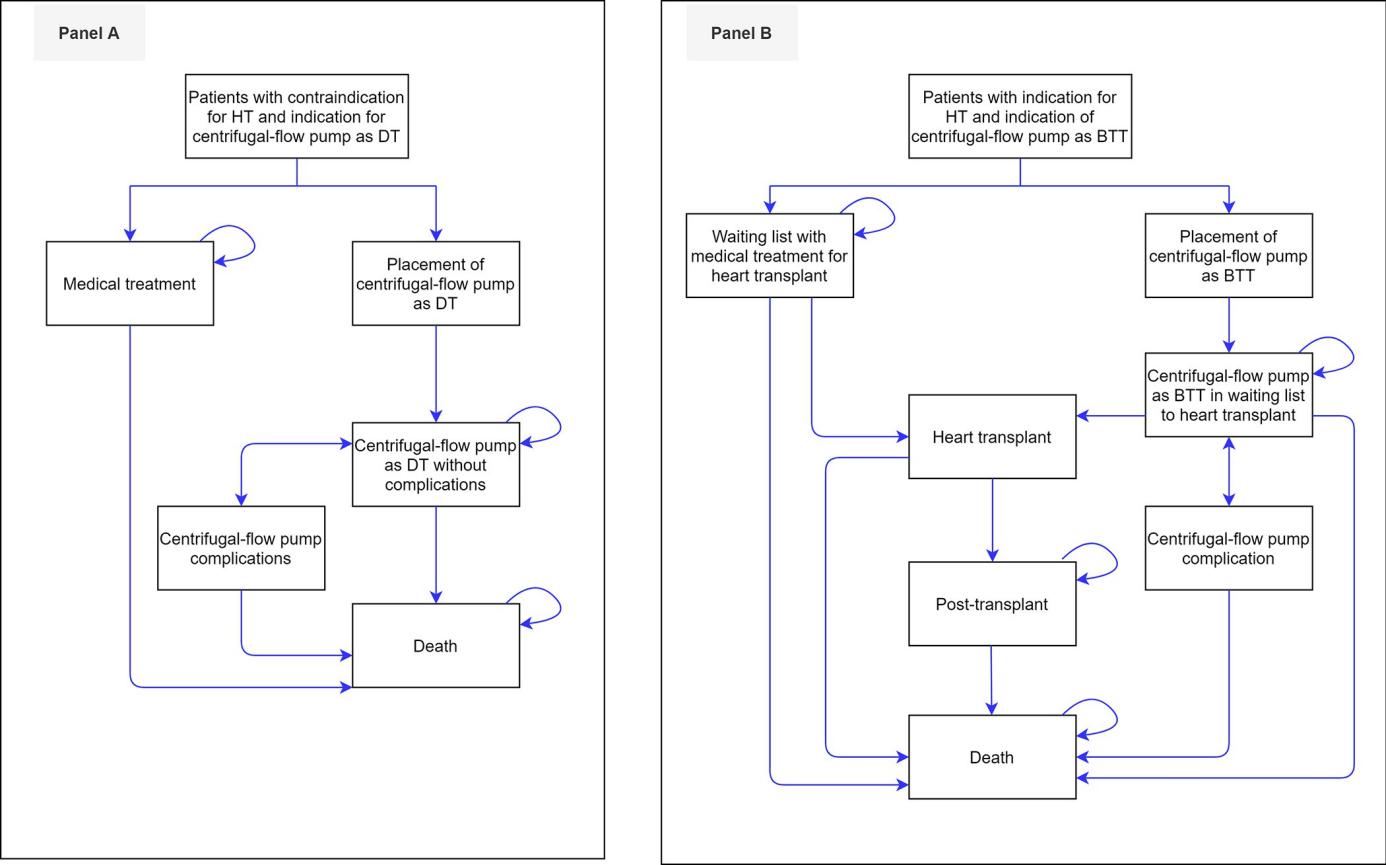
Panel A, Markov structure for Model 1, Panel B, Markov structure for Model 2. Abbreviations. *BTT*, bridge to transplant; *DT*, destination therapy.

For the centrifugal-flow pump as DT, all patients entered have a contraindication for heart transplantation and are candidates for a centrifugal-flow pump as DT. Then patients are grouped into two cohorts. The control cohort is assigned to the “medical treatment” health state whilst the intervention cohort is assigned to the “placement of centrifugal-flow pump as DT” health state. In the control cohort, patients may remain in the “medical treatment” health state or transition to the “death” health state governed by the mortality model. In the intervention cohort, patients start at the “placement of centrifugal-flow pump as DT” state accruing costs involving the surgical implantation of the centrifugal-flow pump and its complications. Afterwards, patients transition to the “Centrifugal-flow pump as DT without complication” health state. Patients can remain in this health state, or transition to the “Centrifugal-flow pump complications”, and after one cycle, return to the “Centrifugal-flow pump as DT without complication” health state, or they can transition to the “death” health state (Fig 1, panel A).

For a centrifugal-flow pump as BTT, patients have an indication for heart transplant plus centrifugal-flow pump as BTT. Patients are grouped either into a control or an intervention cohort. In the control cohort, patients are put on a “waiting list with medical treatment for a heart transplant” and may remain in the current health state, or transition to the “death” health state governed by the mortality model. If the patient survives the mean waiting time for heart transplants, then he/she transitions to the “transplant” health state. In this health state, the patient can either die during heart transplantation and transition to the “death” health state or survive and transition to the “post-transplant” health state. In the intervention cohort, patients start at the “placement of Centrifugal-flow pump as BTT” accruing costs involving the surgical implantation of the centrifugal-flow pump and its complications. Afterwards, patients transition to the “Centrifugal-flow pump as BTT waiting list for a heart transplant” health state. Patients may remain in this latter health state or, as they are at risk of dying or suffering complications caused by the device, patients can transition to the “death” or “Centrifugal-flow pump complication” health state. Patients in the “Centrifugal-flow pump complication” health state can either die or return to the waiting list for heart transplantation (Fig 1, panel B).

Each cycle in both models (i.e. cycle length) lasts one month, during which patients can have clinical events, different utilities, and incur in direct medical costs (such as device placement, routine care, monitoring, hospitalizations, complications). The cycles included in the model were one month long as defined by previous economic evaluations [36–38] and by the fact that during this time frame the patients face high mortality and morbidity rates. Death is an absorbing health state in these models.

### Epidemiological, clinical, and health-related quality of life parameters

We conducted a literature search in scientific literature databases such as PubMed and LILACS (search strategies are available in the S1 File) to retrieve evidence on epidemiological, clinical, and preference-utility values depicting health-related quality of life (HRQoL). When necessary, information was sought by expert consultation, consultation in national official sites such as the Ministry of Health of the Nation, the National System of Information of Procurement and Transplantation of the Argentine Republic (SINTRA) and the Unique Central National Coordination Institute for Ablation and Implant (INCUCAI by its acronym in Spanish) [39]. All the parameters used for the base case analysis and in the sensitivity analysis are described in Table 1.

**Table 1:** Input clinical, epidemiological and utility parameters for both models.

Parameters	Base case (variability range)*	Utility value (standard error)*
Model 1. Clinical and epidemiological parameters		
Mean age of HF patients with contraindication for HT	59 (47 to 71)^35,37–39^	---
Monthly mortality rate of patients in standard medical treatment, INTERMACS 1–4	0.106 (0.079 to 0.132)^19^	0.53 (0.135)^45^
Model 2. Clinical and epidemiological parameters		
Mean age of HF patients without contraindication for HT	51 (39 to 63)^35,37–39^	---
Mean waiting time for HT, in months	3.05 (2 to 9)^22,37^	---
Mean waiting time for HT with centrifugal-flow pump as BTT, in months	6.07 (3.05 to 12)^37^	---
Risk of mortality for HT	0.074 (0.055 to 0.092)^37,42,43^	---
Risk of mortality for post-HT	0.0024 (0.0018 to 0.0030)^37,42,43^	---
Centrifugal-flow pump mortality parameters		
Risk of mortality for centrifugal-flow pump (day 1 to 30)	0.06 (0.045 to 0.075)^39^	---
Risk of mortality for centrifugal-flow pump (month 2 to 3)	0.025 (0.019 to 0.032)^39^	---
Risk of mortality for centrifugal-flow pump (month 4 to 6)	0.013 (0.010 to 0.017)^39^	---
Risk of mortality for centrifugal-flow pump (month 7 to 12)	0.010 (0.008 to 0.013)^39^	---
Risk of mortality for centrifugal-flow pump (month 13 to 24)	0.008 (0.0075 to 0.019)^39^	---
Risk of mortality for centrifugal-flow pump (month 25 to 36)	0.008 (0.006 to 0.011)^39^	---
Risk of mortality for centrifugal-flow pump (month 37 to 48)	0.007 (0.006 to 0.009)^39^	---
Risk of mortality for centrifugal-flow pump (month 49 to 60)	0.006 (0.004 to 0.007)^39^	---
Centrifugal-flow pump monthly rate of complications		
Gastrointestinal bleeding (< = 1 year)	0.016 (0.012 to 0.020)^21,40^	0.60 (0.153)^34^
Gastrointestinal bleeding (>1 year)	0.005 (0.003 to 0.006)^21,40^	0.60 (0.153)^34^
Failure and pump replacement	0.0008 (0.0006 to 0.001)^21,40^	0.53 (0.135)^34^
Disabling stroke (< = 1 month)	0.003 (0.002 to 0.004)^21,40,41^	0.44 (0.112)^48,49^
Disabling stroke (>1 month)	0.0011 (0.0007 to 0.0013)^21,40,41^	0.44 (0.112)^48,49^
Non-disabling stroke (<1 month)	0.037 (0.027 to 0.046)^21,40,41^	0.71 (0.181)^48,49^
Non-disabling stroke (month 1 to 6)	0.038 (0.028 to 0.047)^21,40,41^	0.71 (0.181)^48,49^
Non-disabling stroke (>6 months)	0.030 (0.022 to 0.037)^21,40,41^	0.71 (0.181)^48,49^
Driveline infection (< = 1 year)	0.015 (0.011 to 0.019)^21,40^	0.60 (0.153)^34^
Driveline infection (>1 year)	0.004 to (0.003 to 0.006)^21,40^	0.60 (0.153)^34^
Right heart failure (< = 1 year)a	0.024 (0.018 to 0.030)^21,40^	0.53 (0.135)
Right heart failure (>1 year)a	0.007 to (0.005 to 0.009)^21,40^	0.53 (0.135)
Sepsis (< = 1 year)	0.010 (0.007 to 0.012)^21,40^	0.47 (0.120)^54^
Sepsis (>1 year)	0.003 (0.002 to 0.004)^21,40^	0.47 (0.120)^54^
Ventricular arrhythmia (< = 1 year)	0.014 (0.010 to 0.017)^21,40^	-0.02 (0.005)^53^ b
Ventricular arrhythmia (>1 year)	0.004 (0.003 to 0.005)^21,40^	-0.02 (0.005)^53^ b
HT monthly rate of complication		
Transplant rejection	0.011^43,44^	0.76 (0.193)^34^
Cardiac allograft vasculopathy	0.005^43,44^	0.76 (0.193)^34^
Skin malignancy	0.001^43,44^	0.65 (0.175)^50^
Lymphoma	0.0001^43,44^	0.55 (0.137)^51^
Renal dysfunction with dialysis	0.003^43,44^	0.57^52^
Sepsis	0.009^43,44^	0.47 (0.120)^54^
Other parameters		
Utility of centrifugal-flow pump < = 1 months	---	0.51 (130)^46^
Utility of centrifugal-flow pump >1 months	---	0.72 (0.184)^47^
Utility of stable HT	---	0.76 (0.193)^47^

Notes: a, utility value obtained through expert consultation; b, Disutilities.

Abbreviations. HF, heart failure; HT, heart transplantation.

*References for the sources of the parameters are in brackets.

Patients were registered at ages 59 and 51 in models 1 and 2, respectively. The age difference between cohorts was based on data collected from local registries (SINTRA and INCUCAI) [39], international registries (MEDAMACS [40] and INTERMACS registries [41]), and previous economic evaluations and validated through expert consultation [37]. For patients with a primary indication for a heart transplant, a period of 3.05 month waiting time was assumed, which is the mean waiting time for patients on the emergency/urgency list in Argentina [39]. This decision was based on the guidelines and regulations for heart transplants in Argentina, where it is explicit that hospitalized inotrope/dependent patients and/or those having emergency mechanical (cardiac or pulmonary) assistance devices are considered candidates on the emergency/urgency waiting list, while those who do not meet these criteria are considered elective candidates [24]. For patients with BTT, the average waiting time was set at 6.7 months based on the mean waiting time for patients on the elective heart transplant list in Argentina (SINTRA-INCUCAI) [39]. For those patients who had received the centrifugal-flow pump as BTT, we assumed that they would wait on an outpatient basis, and they could thus be transplanted electively. This assumption was validated by local experts in advanced HF and heart transplant.

We assume that patients with clinical evolution of advanced HF and contraindication for heart transplantation remain under inotropic-dependent hospitalization for 25% of their surviving lifetime, while the remaining time is under close outpatient medical follow-up. This assumption is based on the REMATCH study that enrolled patients with contraindication for heart transplantation and indication for medical management or placement of LVAD as DT [19]. The monthly mortality rate calculated for the standard medical treatment was obtained from the survival data from the medical management branch of the REMATCH study, and it was later validated by expert consultation [19]. On the other hand, mortality calculated for patients with LVAD came from the INTERMACS registry [41]. The registry includes 1292 patients with LVAD (including centrifugal-flow pump) enrolled during 2014 and 2019 and reports 60-month follow-up survival data according to indication subgroups (DT or BTT). The registry was also used to calculate the mortality of uncomplicated transplant patients, since it includes data on transplanted patients from 1982 to this date, and in its latest report (2010–2017, *n* = 31938) it shows survival data after more than 10 years of follow-up [41]. This registry receives information from multiple national and international centers, including INCUCAI from Argentina [39].

We modelled the complications associated with the device as reported by Mehra and colleagues in the MOMENTUM 3 study [42]. It was decided to consider those with the greatest frequency of appearance and the greatest prognostic impact. To model the occurrence of stroke (disabling and non-disabling stroke) we used the data published by Colombo and colleagues regarding the characteristics of stroke in patients enrolled in the MOMENTUM-3 study (sub-analysis of the main study), which showed a marked increase in the frequency of the event during the first month after placement of the device [43]. For the rest of the complications caused by the centrifugal-flow pump, during the first year after placement we took an approach that arose from the statistical analysis of adverse event curves during follow-up (further details are reported in the MOMENTUM 3 Supplementary Appendix) [21].

The risk of mortality for heart transplant recipients and the risk of mortality post heart transplantation were defined based on the data from the International Society for Heart and Lung Transplantation (ISHLT) [44,45] and from INCUCAI [39]. We considered the complications most frequently reported in the local setting. The sources of the complication rate were a prospective cohort of 333 heart transplant patients at the Favaloro Foundation in Argentina, between 1993 and 2009 [46], and international data published by ISHLT in 2019 [45].

The HRQoL-utility values for patients under standard medical management are based on a study that measures utilities by using the Standard Gamble Technique in inotrope-dependent patients with advanced HF, who are eligible for an LVAD [47]. For patients with centrifugal-flow pump, utilities were obtained from an extrapolation of continuous-flow device utilities. This assumption is based on two main facts. First, to date there is no published data regarding HRQoL for patients with centrifugal-flow pump. Second, in the MOMENTUM 3 study, no significant differences were found between the continuous-flow device and the centrifugal-flow pump groups using several instruments and weights (European Quality of Life–5 Dimensions questionnaire (EQ‐5D‐5L) visual analogue scale (VAS) score over time, the EQ-5D5L total score over time, and the Kansas City Cardiomyopathy Questionnaire (KCCQ)-9 [21]. Consequently, the utilities for patients during the first month of LVAD were based on the study of Sharples and colleagues who evaluated utilities in the first month of LVAD placement in patients with indication for LVAD as BTT [48]. After one month, utilities were based on the Seventh INTERMACS Annual Report [49]. This report was also used to obtain utilities for stable heart transplant patients.

Utilities for disabling and non-disabling stroke were adjusted according to the type of event following the modified Rankin classification for stroke, a scale used in the MOMENTUM 3 study to differentiate disabling stroke (Rankin 4–5) and non-disabling stroke (Rankin 2–3) [21]. The utilities were retrieved from a systematic review of utilities in patients with stroke [50,51]. For the rest of the complications due to the centrifugal-flow pump and heart transplant, we retrieved utilities from systematic reviews [52–54] and previous economic evaluations [36,55,56]. When necessary, utility values were adapted to the local context using expert consultation.

### Cost parameters

The micro-costing approach was used to estimate direct medical costs from the payer’s perspective (SS and PS). Identification and quantification of medical resources were made through literature review, validated by local clinical expert’ consultation, whilst cost estimations were made using the Healthcare Cost Database of the Institute for Clinical Effectiveness and Health Policy [57]. This database contains cost information based on the nomenclature of medical practices for SS and PS. Costs were inflated to December 2019 Argentinian pesos (exchange rate ARS $ 1 = US $ 59.95).

The centrifugal-flow pump device purchase price provided by the manufacturer (for HeartMate 3) was ARS $ 7 104 276. Pharmacological treatment in the outpatient/stable cycle was based on the local clinical practice guidelines [24] and standard HF healthcare resources utilization were estimated according to the local clinical practice guidelines for the treatment of HF [24]. The identification and quantification of the resources used during HF hospital stay in Argentina were based on two local studies [58,59]. The parameters of the direct medical costs used in the model are presented in the S2 File.

### Sensitivity analyses

To evaluate individual parameter uncertainty, a one-way deterministic sensitivity analysis was performed of each parameter included in the model. We also performed a probabilistic sensitivity analysis to assess multiple parameter uncertainty, in which in 1000 iterations (Second Order Monte Carlo Simulation) different cost-effectiveness estimates are simulated by assigning a specific probability distribution to each of the variables considered in the model. The results were reported graphically through tornado diagrams (deterministic analysis), acceptability curves, and cost-effectiveness plans (probabilistic analysis). For Model 2 we additionally performed an alternative scenario analysis considering variabilities in time on the waiting list for heart transplantation. In this additional scenario, we assumed equal waiting intervals for both patients with centrifugal-flow pump as BTT and patients with heart transplants.

## Results

### Base case results

For both models, base case results are reported in Table 2. At a discount rate of 5%, the centrifugal-flow pump as DT increased QALY in the intervention cohort in comparison to the control cohort by 3.5. The centrifugal-flow pump also increased costs compared to standard medical management by ARS $ 8,059,899 in the Social Security perspective and by ARS $ 8,097,092 in the Private Sector perspective. The resultant ICERs were ARS $ 2,299,352 and ARS $ 2,309,937 for the Social Security and Private sectors, respectively. In the base case results with no discount rate, the resultant ICERs were ARS $ 1,673,990 and ARS $ 1,706,001 for the Social Security and Private Sectors, respectively.

**Table 2:** Base case results.

Model	Strategy	Per patient cumulative costs (ARS $)		Incremental costs (ARS $)		Per patient cumulative QALY	Incremental QALY	Incremental cost-effectiveness ratio (ARS $ per QALY gained)*	
		Social Security	Private Sector	Social Security	Private Sector			Social Security	Private Sector
Centrifugal-flow pump in contraindication of heart transplant	Base case results with 5% discount rate								
	Standard medical management	1,441,544	1,976,601			0.399			
	Centrifugal-flow pump as destination therapy	9,501,533	10,073,693	8,059,989	8,097,092	3.904	3.505	2,299,352	2,309,937
	Base case results without discount rate								
	Standard medical management	1,496,547	2,052,021			0.414			
	Centrifugal-flow pump as destination therapy	10,003,635	10,721,785	8,507,088	8,669,764	5.496	5.082	1,673,990	1,706,001
Centrifugal-flow pump as bridge-to-transplant	Base case results with 5% discount rate								
	Standard medical management	4,871,487	5,617,656			6.16			
	Centrifugal-flow pump as bridge to transplant	13,091,756	14,014,811	8,220,269	8,397,155	6.90	0.74	11,159,488	11,399,621
	Base case results without discount rate								
	Standard medical management	7,036,901	8,005,844			10.61			
	Centrifugal-flow pump as bridge to transplant	15,512,380	16,714,801	8,475,479	8,708,957	11.85	1.24	6,838,430	7,026,811

Abbreviations. QALY, quality-adjusted life years. Exchange rate USD 1 = ARS 59.95.

* 1 Gross Domestic Product per capita equivalent to ARS $700 473.

At a discount rate of 5%, the centrifugal-flow pump as BTT increased QALY in the intervention cohort in comparison to the control cohort by 0.74. Costs also increased in the intervention cohort in comparison to the control cohort by ARS $ 8,220,269 and ARS $ 8,397,155 in the Social Security perspective and the Private Sector perspective, respectively. The resultant ICERs were ARS $ 11,159,488 for the Social Security perspective and ARS $ 11,399,621 for the Private Sector perspective. In the base case results with no discount rate, the resultant ICERs were ARS $ 6,838,430 and ARS $ 7,026,811 for the Social Security and the Private Sector perspective, respectively.

### Sensitivity analysis and additional scenarios

**Fig 2:**
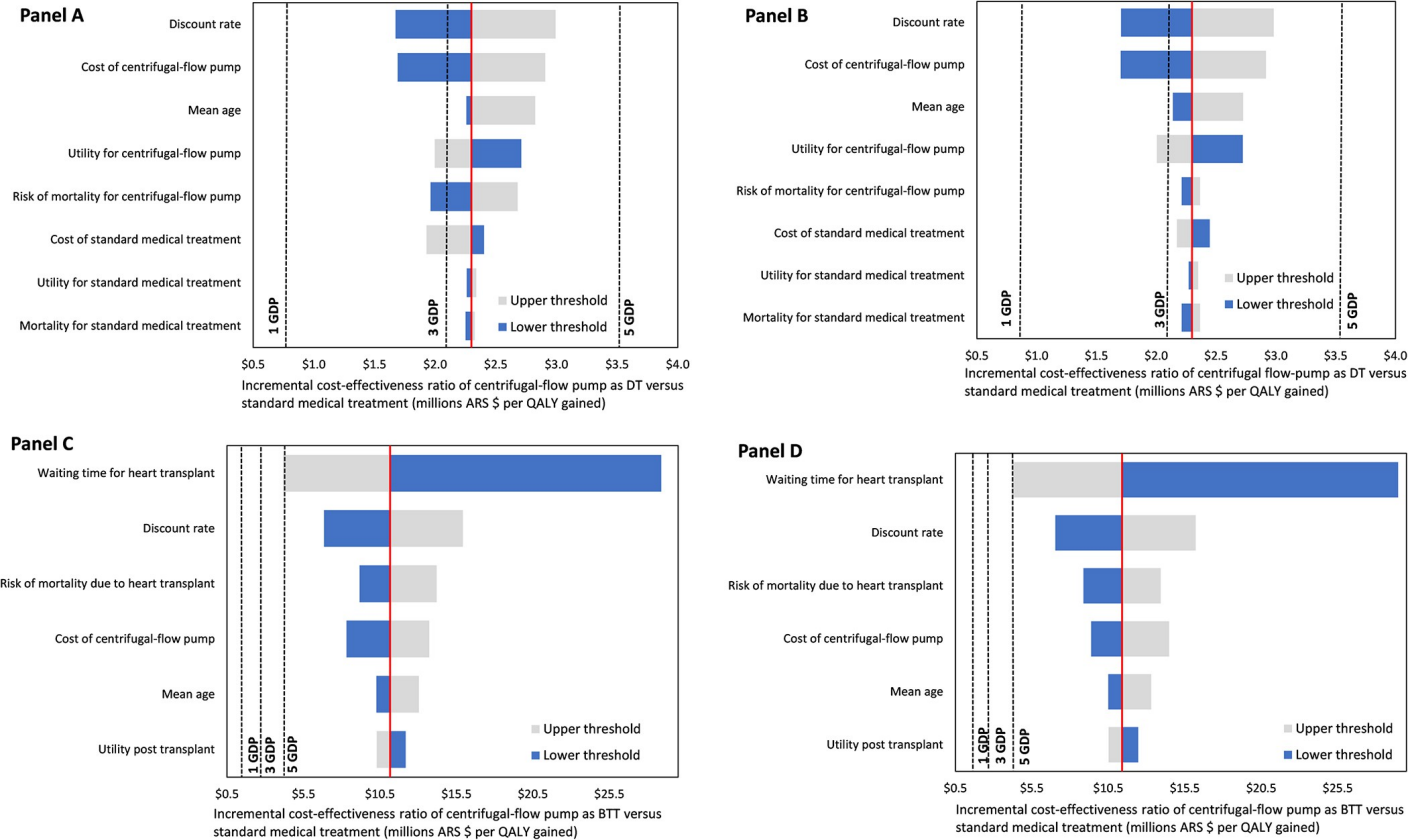
Tornado diagram: Series of n-way sensitivity analysis of the centrifugal-flow pump as destination therapy (DT) or bridge-to-transplant (BTT). For Model 1 (DT), results are reported in Panels A and B for the Social Security and Private Sector perspectives, respectively. For Model 2 (BTT), results are reported in Panels A and B for the Social Security and Private Sector perspectives, respectively. Notes. The bars indicate the range of ARS $ per QALY obtained with the centrifugal-flow pump as DT or BTT compared to standard medical management via 1-way sensitivity analyses of the input parameters across the range of values. The solid red line represents the base case cost-effectiveness result of ARS $ 2,299,352 per QALY gained for the Social Security perspective and ARS $ 2,309,937 for the Private Sector perspective. Exchange rate USD 1 = ARS 59.95.

The one-way sensitivity analysis of the centrifugal-flow pump as DT is presented in Fig 2. Panels A and B represent the Social Security and Private Sector perspectives, respectively. In Panel A, the ICER was most sensitive to the discount rate and the cost of the centrifugal-flow pump. When we increased the discount rate to 10%, the resultant ICER was ARS $ 2,993,269. On the other hand, when we increased the price of the centrifugal flow pump by 25%, the resultant ICER was 2,907,364. However, reducing the price of the centrifugal-flow pump yielded an ICER of ARS $ 1,691,340. These results in Panel B are quite similar to the results for the Private Sector perspective. For both perspectives, other parameters included in the model have a lower impact on the ICERs.

The one-way sensitivity analysis of the centrifugal-flow pump as BTT is displayed in Panel C (Social Security perspective) and Panel D (Private Sector perspective). For both perspectives, the parameters that most affected ICERs were the waiting time for a heart transplant, the discount rate, the risk of mortality due to heart transplant and the cost of the centrifugal-flow pump. In the alternative scenario analysis, which considered variabilities in the waiting time for heart transplant, when we reduced the waiting time to 1.5 months, the resulting ICERs were ARS $ 28,896,398 and ARS $ 29,457,500 for the Social Security and Private Sector perspectives, respectively. When we increased the waiting time to 9 months, the resulting ICERs were ARS $ 4,175,245 and ARS $ 4,266,036 for the Social Security and Private Sector perspectives, respectively.

**Fig 3:**
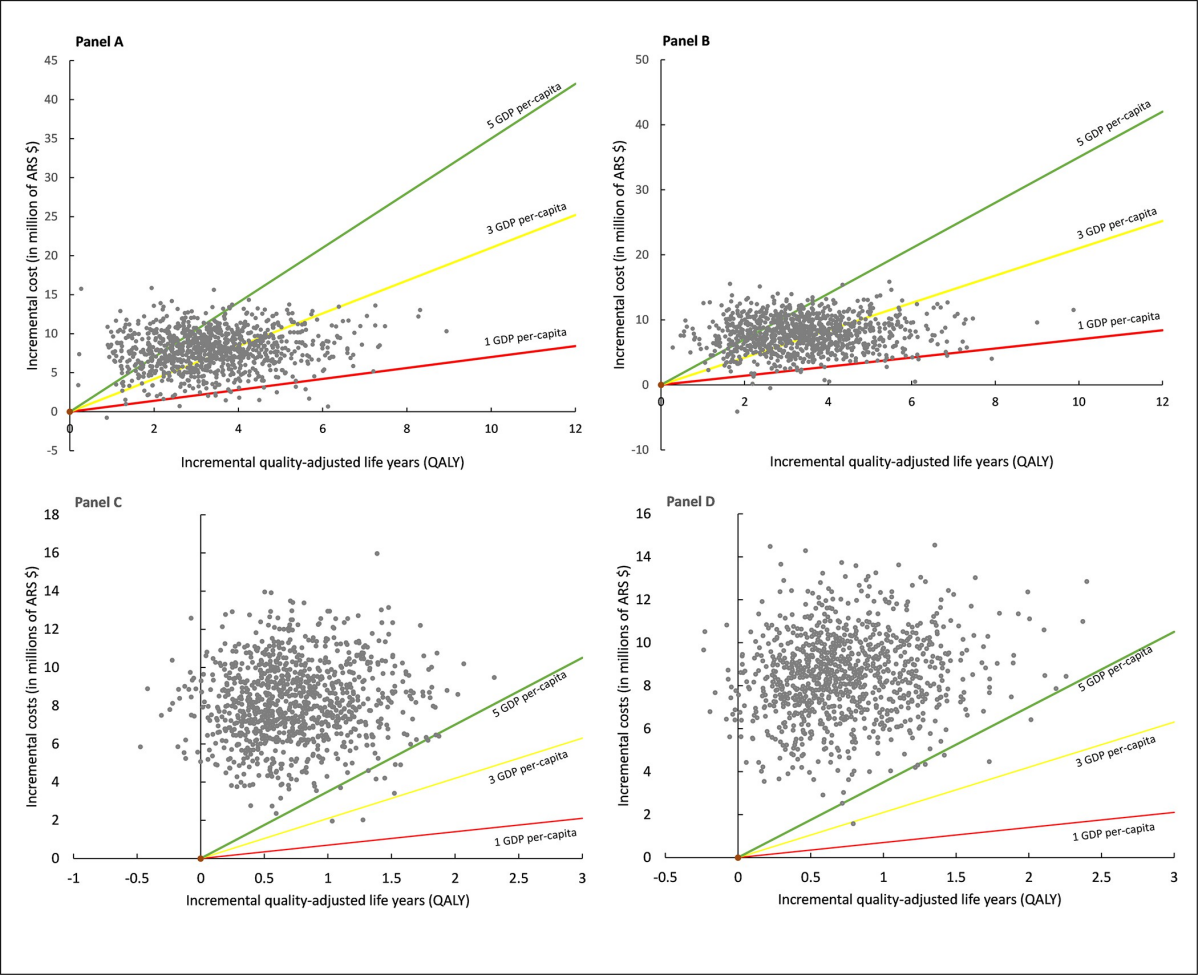
Incremental cost-effectiveness scatter plot for Model 1: Centrifugal-flow pump as a destination therapy in patients with contraindication for heart transplantation versus standard medical management. Panel A displays the results for the Social Security perspective and Panel B displays results for the Private Sector perspective. The incremental cost-effectiveness scatter plots for Model 2 (centrifugal-flow pump as bridge-to-transplant versus a heart transplant as treatment) are reported in Panel C (Social Security perspective) and Panel B (Private Sector perspective).

The incremental cost-effectiveness scatter plots for the centrifugal-flow pump as DT or BTT are reported in Fig 3. For Model 1 (DT), the results are reported in Panel A (Social Security perspective) and Panel B (Private Sector perspective). For both perspectives, the centrifugal-flow pump as DT was more expensive and yielded more QALYs in comparison to the standard medical management, and all the points thus fell into the Northeast quadrant of the cost-effectiveness plane. Most of the points are above the willingness to pay threshold of 1 GDP per-capita in both perspectives, but most of the points are between the willingness to pay thresholds of 3 and 5 GDP per-capita. For Model 2 (BTT), the incremental cost-effectiveness scatter plots are reported in Panel C (Social Security perspective) and Panel D (Private Sector perspective). In some points, for both perspectives there were cases in which heart transplantation as a treatment had higher QALYs in comparison to the centrifugal-flow pump as BTT. However, the bulk of the points are located in the Northeast quadrant of the cost-effectiveness plane. Most of the points are above the willingness to pay threshold of 5 GDP per-capita.

**Fig 4:**
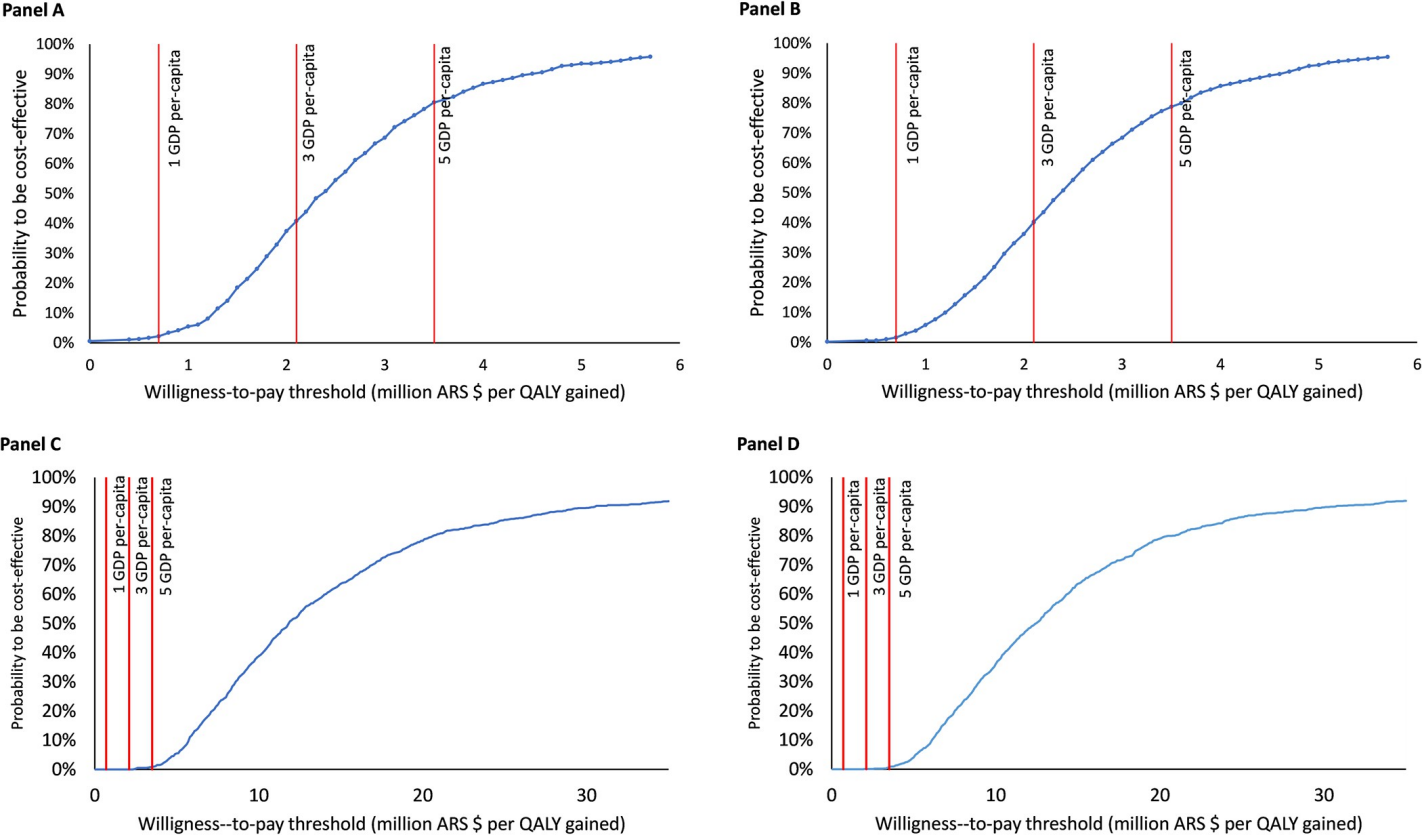
Cost-effectiveness acceptability curves for the centrifugal-flow pump as destination therapy (DT). Results for Social Security and Private Sector perspectives are displayed in Panel A and Panel B. The cost-effectiveness acceptability curves for a centrifugal-flow pump as bridge-to-transplant (BTT) are reported in Panel C (Social Security perspective) and Panel D (Private Sector perspective).

In Argentina, as willingness-to-pay among third-party payers from different sectors may be heterogeneous, we assessed our findings using several potential thresholds of willingness-to pay in both models. The cost-effectiveness acceptability curves for the centrifugal-flow pump as DT or BTT are reported in Fig 4. For Model 1 (DT) and for the Social Security perspective, the probability that the centrifugal-flow pump as DT be cost-effective is 2.3%, 39.8% and 80.9% at one, three, and five GDP per-capita, respectively (GDP = ARS $ 700 473) (Panel A). For the Private Sector perspective, the probabilities are 1.7%, 38.8%, and 79%, respectively (Panel B). On the other hand, the cost-effectiveness acceptability curve for the centrifugal-flow pump as BTT is displayed in Panel C (Social Security perspective) and Panel D (Private Sector perspective). For the Social Security perspective, the probability that the Centrifugal-flow pump as BTT be cost-effective is 0%, 0%, and 0.9% at one, three, and five GDP per-capita, respectively. For the Private Sector perspective, the probabilities are 0%, 0.1%, and 0.7%, respectively.

## Discussion

This study aimed to estimate the cost-effectiveness of the novel LVAD type centrifugal-flow pump, as DT or BTT treatment strategy in patients with advanced HF. As DT, our analysis suggests that patients who are inotropic-dependent and with contraindication for a heart transplant, if treated with the centrifugal-flow pump rather than by standard medical management, would live longer and have an improved quality of life, but they would also incur in additional healthcare costs. This implies an ICER equivalent to ARS $ 2,299,352 and ARS $ 2,309,937 from a Social Security and Private Sector perspectives, which are values between 3 and 5 GDP per-capita in Argentina. The interpretation of these results for the Centrifugal-flow pump as DT, and the implications for decision-making, are not straightforward since there is no current explicit cost-effectiveness threshold in Argentina. In turn, the willingness to pay for QALY could vary between health sub sectors within the country. In a recent estimation performed by the Argentine Government Health Office, 41.8% of the total expenditure in health corresponds to the SS, while 29.5% corresponds to the PS and the remaining fraction corresponds to the public sector [60]. Thus, the final decision to determine its cost-effectiveness will depend on the willingness-to-pay for QALYs by decision makers from each health subsector in Argentina, also taking into account that health care systems may accept higher thresholds than usual in some cases of high priority and non-highly prevalent patient groups, as could be this case. Considering the impact of the centrifugal-flow pump on the survival rate and the quality of life in critically ill patients with contraindication for heart transplant, providing access to therapies such as the new generation of LVAD as DT could become, in certain circumstances, a valuable treatment strategy to significantly improve health outcomes.

When we examined the uncertainty about the estimates for DT, our conclusion remained stable, consistently obtaining values below a willingness-to-pay threshold of 5 GDP per-capita. In previous analyses, the first and second generations of LVAD were not found to be cost-effective compared to medical management in the population ineligible for a heart transplant [38,61]. The better cost-effectiveness results of the centrifugal-flow pump LVAD in comparison to the previous generation of LVAD may be attributed to its superiority in terms of survival free of disabling stroke or reoperation to replace or remove a malfunctioning device [21,42].

Results were quite different for BTT. For patients with an indication for heart transplant, our analysis showed that the centrifugal-flow pump, as compared to a heart transplant, has a larger increase in cost per QALY, with an ICER equivalent to approximately 16 GDP per-capita, a result very unlikely to be considered cost-effective, even in the private sector. This result is similar to the analysis performed by Long and colleagues, who assessed the cost-effectiveness of the second generation of LVAD in the United States [36]. Another similarity between our study and the one performed by Long and colleagues is the approach used to account for the decrement in utility from complications, by stratifying patients based on the INTERMACS classification for patients with advanced HF [35].

In the sensitivity analysis for BTT lower ICERs were found when we increased the waiting time by 9 months, and thus this parameter had a considerable impact on ICER. This is a relevant issue since organ shortage and the increasing prevalence of HF may prolong the waiting time in the future, for example during this COVID-19 pandemic situation the availability of organs has been limited. Future models may include additional information, for example information about the intensity of inpatient rehabilitation [62,63], to better elucidate the impact of longer waiting times on the health outcomes and on the ICERs.

### Strengths and limitations

Our analysis rested upon recognized modelling techniques and used large and comprehensive databases with information from local patients [39,46]. When no local information was available, large datasets from international patients were used and when necessary, they were adapted to the local context by expert consultation. However, our study has some limitations to note. Studying the population in advanced stages of HF is challenging due to the high heterogeneity in patients. A previous study had found that the cost-effectiveness of a particular treatment highly relies on the population who receives it [64]. Consequently, it is important to identify the subgroup of population that will derive benefits based on an evidence-based selection. In this study, to reduce selection bias, we established clear inclusion criteria based on the severity of the symptoms [34], the INTERMACS classification for patients with advanced HF [35], and took transplant eligibility into account. A limitation of this study is that demographic and non-cardiovascular clinical characteristics were not considered in the modelling strategy. For example, a systematic review found that, in comparison to men, women are at greater risk of significant complications after CF-LVAD implantation [65]. Another aspect to consider is that our models simplify the complex progression and management of the evaluated patients, assuming that the costs and lower quality of life due to complications would be constant during follow-up and that the survival of patients with the implanted device would be similar to that reported in international registries. However, a systematic examination and responsible reporting of uncertainty regarding the parameters included in the models were made based on a recognized guideline [66]. Lastly, this study considers only the costs of medical treatment and the HRQoL for patients. Despite the evidence that shows the impact of LVAD implantation on the quality of life of caregivers [67], this is rarely quantified mainly due to lack of data. Future research should include additional benefits in the treatment with the centrifugal-flow pump to re-evaluate the cost-effectiveness and inform decision-makers based on a wider perspective.

### Conclusions

The LVAD type centrifugal-flow pump prolongs life and improves the quality of life at a higher cost. In patients who are ineligible for heart transplant the ICER was between 3 and 5 GDP per capita, and as bridge-to-transplant it was above 15 GDP, but highly dependent on waiting times for transplantation. Nevertheless, the centrifugal-flow pump as destination therapy has always been significantly more cost-effective than as bridge-to-transplant. Since in Argentina there is no current explicit cost-effectiveness threshold, the final decision on reimbursement will depend on the willingness to pay for QALYs in each health subsector.

## Supporting information

S1 Table Search strategies used in PubMed and LILACS. (DOCX)

S2 Table Cost input parameters, in Argentine pesos (ARS $ December 2019). Exchange rate USD 1 = ARS 59.95 ^a^ To estimate the minimum and maximum values, the value of the centrifugal-flow pump was varied by ± 25% of its base value. ^b^ The cost includes the weighted acute complications of a heart transplant during the surgical process.
(DOCX)

S1 File Model for destination therapy. (XLSM)

S2 File Model for bridge to transplant. (XLSM)
