# Supplementary material for: Cost-effectiveness of a centrifugal-flow pump for patients with advanced heart failure in Argentina
Source: PLoS One. 2022 Aug 1;17(8):e0271519. doi: 10.1371/journal.pone.0271519 (PMC9342761; doi:10.1371/journal.pone.0271519)
Supplement: S1 Table — (DOCX) [file pone.0271519.s001.docx]

**Supporting Information S1**. Search strategies used in PubMed and LILACS.

| **Database** | **Strategy** |
| --- | --- |
| **PubMed** | ((HeartMate[tiab] OR (Left Ventricular*[tiab] OR Ventricular Assist*[tiab] OR Artificial Heart*[tiab] OR Heart Assist Device*[tiab] OR centrifugal flow-pump[tiab]) AND (Advance Heart Failure[tiab] OR Heart Failure*[tiab] OR Heart Transplant*[tiab])). |
| **LILACS** | (tw:(HeartMate)) AND (tw:(Heart Decompensation OR Heart Failure OR Myocardial Failure OR Left Sided Heart Failure OR Cardiac Failure OR Congestive Heart Failure OR Advance Heart Failure OR Heart Transplant)) |
