# Supplementary material for: Cost-effectiveness of a centrifugal-flow pump for patients with advanced heart failure in Argentina
Source: PLoS One. 2022 Aug 1;17(8):e0271519. doi: 10.1371/journal.pone.0271519 (PMC9342761; doi:10.1371/journal.pone.0271519)
Supplement: S2 Table — Exchange rate USD 1 = ARS 59.95 a To estimate the minimum and maximum values, the value of the centrifugal-flow pump was varied by ± 25% of its base value. b The cost includes the weighted acute complications of a heart transplant during the surgical process. (DOCX) [file pone.0271519.s002.docx]

**Supporting Information S2**. Cost input parameters, in Argentine pesos (ARS $ December 2019)

|  | **Social security (ARS $)** | | |  | **Private sector (ARS $)** | | |
| --- | --- | --- | --- | --- | --- | --- | --- |
| **State or adverse event** | **Minimum** | **Mean** | **Maximum** |  | **Minimum** | **Mean** | **Maximum** |
| Common cost |  |  |  |  |  |  |  |
| Monthly standard medical treatment inotrope-dependent | 56,492 | 159,720 | 262,847 |  | 165,794 | 219,003 | 272,202 |
| Centrifugal-flow pump |  |  |  |  |  |  |  |
| Centrifugal-flow pump HeartMate 3 + implantation procedure costs ^a^ | 5,953,373 | 8,084,656 | 10,215,939 |  | 6,118,989 | 8,250,272 | 10,381,555 |
| Driveline infection | 133,512 | 283,685 | 433,342 |  | 313,988 | 385,882 | 457,591 |
| Right heart failure | 73,092 | 225,472 | 378,623 |  | 241,085 | 316,225 | 391,054 |
| Stroke | 73,622 | 194,787 | 315,090 |  | 212,372 | 271,267 | 330,104 |
| Monthly follow-up for non-disabling stroke | 22,860 | 28,575 | 34,290 |  | 25,114 | 31,392 | 37,671 |
| Monthly follow-up for disabling stroke | 36,504 | 45,630 | 54,756 |  | 39,632 | 49,540 | 59,448 |
| Gastrointestinal bleeding | 82,545 | 159,487 | 241,777 |  | 155,035 | 201,942 | 243,353 |
| Ventricular arrhythmia | 50,098 | 121,179 | 193,672 |  | 129,240 | 164,136 | 198,888 |
| Sepsis | 194,424 | 409,473 | 553,016 |  | 258,362 | 532,540 | 735,837 |
| Failure and pump replacement | 6,022,525 | 7,909,064 | 9,863,267 |  | 6,676,122 | 8,537,616 | 10,402,765 |
| Monthly follow-up of patients with centrifugal-flow pump | 6,310 | 8,439 | 11,283 |  | 11,825 | 12,335 | 12,958 |
| Heart transplant |  |  |  |  |  |  |  |
| Monthly waiting time list for heart transplant | 61,719 | 164,947 | 268,074 |  | 172,592 | 225,801 | 279,000 |
| Heart transplant ^b^ | 1,594,019 | 1,977,548 | 2,347,029 |  | 2,073,147 | 2,359,903 | 2,644,606 |
| Monthly follow-up for patients with a heart transplant (month 1 to 12) | 28,726 | 30,308 | 32,041 |  | 32,893 | 33,380 | 33,857 |
| Monthly follow-up for patients with a heart transplant (>month 12) | 28,714 | 30,295 | 32,029 |  | 32,880 | 33,368 | 33,844 |

Exchange rate USD 1 = ARS 59.95

^a^ To estimate the minimum and maximum values, the value of the centrifugal-flow pump was varied by ± 25% of its base value.

^b^ The cost includes the weighted acute complications of a heart transplant during the surgical process.
